# Absolute oxygen-guided radiation therapy improves tumor control in three preclinical tumor models

**DOI:** 10.3389/fmed.2023.1269689

**Published:** 2023-10-12

**Authors:** Inna Gertsenshteyn, Boris Epel, Mihai Giurcanu, Eugene Barth, John Lukens, Kayla Hall, Jenipher Flores Martinez, Mellissa Grana, Matthew Maggio, Richard C. Miller, Subramanian V. Sundramoorthy, Martyna Krzykawska-Serda, Erik Pearson, Bulent Aydogan, Ralph R. Weichselbaum, Victor M. Tormyshev, Mrignayani Kotecha, Howard J. Halpern

**Affiliations:** ^1^Department of Radiation and Cellular Oncology, The University of Chicago, Chicago, IL, United States; ^2^Department of Radiology, The University of Chicago, Chicago, IL, United States; ^3^Center for EPR Imaging In Vivo Physiology, The University of Chicago, Chicago, IL, United States; ^4^O2M Technologies, Chicago, IL, United States; ^5^Department of Public Health Sciences, The University of Chicago, Chicago, IL, United States; ^6^Department of Biophysics and Cancer Biology, Jagiellonian University, Kraków, Poland; ^7^Novosibirsk Institute of Organic Chemistry, Novosibirsk, Russia

**Keywords:** hypoxia, oxygen, electron paramagnetic resonance, preclinical imaging, radiotherapy

## Abstract

**Background:**

Clinical attempts to find benefit from specifically targeting and boosting resistant hypoxic tumor subvolumes have been promising but inconclusive. While a first preclinical murine tumor type showed significant improved control with hypoxic tumor boosts, a more thorough investigation of efficacy from boosting hypoxic subvolumes defined by electron paramagnetic resonance oxygen imaging (EPROI) is necessary. The present study confirms improved hypoxic tumor control results in three different tumor types using a clonogenic assay and explores potential confounding experimental conditions.

**Materials and methods:**

Three murine tumor models were used for multi-modal imaging and radiotherapy: MCa-4 mammary adenocarcinomas, SCC7 squamous cell carcinomas, and FSa fibrosarcomas. Registered T2-weighted MRI tumor boundaries, hypoxia defined by EPROI as pO_2_ ≤ 10 mmHg, and X-RAD 225Cx CT boost boundaries were obtained for all animals. 13 Gy boosts were directed to hypoxic or equal-integral-volume oxygenated tumor regions and monitored for regrowth. Kaplan–Meier survival analysis was used to assess local tumor control probability (LTCP). The Cox proportional hazards model was used to assess the hazard ratio of tumor progression of Hypoxic Boost vs. Oxygenated Boost for each tumor type controlling for experimental confounding variables such as EPROI radiofrequency, tumor volume, hypoxic fraction, and delay between imaging and radiation treatment.

**Results:**

An overall significant increase in LTCP from Hypoxia Boost vs. Oxygenated Boost treatments was observed in the full group of three tumor types (*p* < 0.0001). The effects of tumor volume and hypoxic fraction on LTCP were dependent on tumor type. The delay between imaging and boost treatments did not have a significant effect on LTCP for all tumor types.

**Conclusion:**

This study confirms that EPROI locates resistant tumor hypoxic regions for radiation boost, increasing clonogenic LTCP, with potential enhanced therapeutic index in three tumor types. Preclinical absolute EPROI may provide correction for clinical hypoxia images using additional clinical physiologic MRI.

## 1. Introduction

Hypoxic resistance in living systems to X-ray radiation has been known for over a century (1, 2). Observation of resistant hypoxic rims in human lung cancer (3) led to an enormous effort to eliminate the hypoxic compartment. The British Hyperbaric Oxygen Trials in the 1970s (4) and trials of administration of oxygen mimetic nitroimidazole hypoxic sensitizers in the 1980s and 1990s showed indications of enhanced efficacy (5), but difficulties in radiation delivery with hyperbaric oxygen and the toxicity of sensitizers has diminished enthusiasm for application. These trials assumed that all tumors have clinically significant hypoxia (6).

Multiple oxygen partial pressure (pO_2_) measurements with Eppendorf needle electrodes were performed in the 1990s and 2000s in various tumors (7–9). These studies found that tumors with higher hypoxic fraction measurements (most often with a threshold defined as median or mean pO_2_ ≤ 10 mmHg) were significantly more likely to fail radiation treatment. This established hypoxia as both a prognostic biomarker for radiation resistance and predictive of tumor control (10). However, it provided little insight into the local pattern of the hypoxia. Was hypoxic resistance sufficiently localized to tumor subvolumes that could be treated with a radiation boost?

Meanwhile, we have witnessed the birth of the field of radiomics, the extraction of multiple quantifiable, classifiable features of medical images that correlate with disease features, processes, and response to therapy. Correlates of these image classifiers include the local genetics, protein composition (proteomics), and microenvironment, particularly tumor microenvironment (11, 12). Two forms of radiomic criteria have been used to define aggressive malignancy: clinical features and generic pattern based features (12).

Gallez recently reviewed the merits of hypoxia image biomarkers to better stratify patients for targeted interventions (13). For example, Mu et al. demonstrated that PET/CT image features could be used to select patients with durable responses to either immune check point inhibitors or tyrosine kinase inhibitors as decision support for treatment in Non-Small Cell Lung Cancer (14). A conclusion from Jardim-Perassi et al. was the consistency of hypoxia with tumor aggressiveness (15). Diepart et al. enhanced preclinical radiation response by reducing O_2_ metabolism with Arsenic Trioxide in tumors (16). Rischin et al. increased tumor control with the hypoxia toxin Tirapazamine in head and neck cancer but found dose limiting toxicity in uterine cervical cancer (17, 18). Ferini et al. reviewed the potential benefit of using oxygen-guided radiation therapy with immunotherapy (19). Finally, and most compelling, have been the studies from Riaz et al. where, among Human Papilloma Virus (HPV) positive patients, those with ^18^F-Misonidazole PET scans showing no hypoxia were controlled with radiation doses of 30 Gy to gross tumor, rather than the conventional 70 Gy (20). Images and their analyses provide crucial insight into general characteristics of malignant tumors and suggested interventional strategies to enhance prognosis and predict outcome. These hypoxia responsive approaches, however, did not provide individualized patient tumor specific locations toward which to focus local therapy to eliminate the last resistant tumor clonogen.

Hypoxia imaging has been developed during the same period as the advancement of radiation technology to deliver highly conformal intensity modulated radiation therapy (IMRT) capable of accurately sculpting radiation doses within the patient to minimize radiation toxicity to organs at risk (OAR). Evidence of advantage from delivery of higher radiation boost doses to hypoxic tumor portions remains both a clinical and preclinical challenge to this day. This is due to a major missing requirement in clinical radiation technology: the ability to image resistant hypoxic tumor subvolumes that require extra radiation dose for control, or determine whether there is insufficient hypoxia to warrant the high radiation doses commonly used to control hypoxic tumors. The avoidance of dose inhomogeneities in the planning treatment volume restricts the dose gradients achievable in the surrounding tissue. With the possibility of detection of oxygen landscape within tumor tissue, the present IMRT can be enhanced by employing its ability to sharpen dose gradients outside the patient treatment volume to spare dose to OARs while boosting any hypoxic subvolumes, thus potentially improving post-treatment health.

To date, several image modalities have identified tumors with qualitatively high and low levels of hypoxia. Early images localizing hypoxia included positron emission tomography (PET) of ^18^F radiolabeled nitroimidazoles, sensitizers that are selectively retained in hypoxic tumor (21) and Blood Oxygen Dependent Level (BOLD) MRI (22–24). Preclinical use of injection of ^19^F hexafluorobenzene injected into a plane of animal tissue provides a pO_2_ quantitation for voxel by voxel comparisons in a limited purview of a tumor, but fails to base evaluation on the entire tumor (25). Oxygen-enhanced MRI (based on ^1^H T1 MRI with both oxygen and air breathing) has been suggested as the basis for potential boost treatment (26) but recent studies suggest further work to be necessary (27).

Clinical attempts at focusing radiation boosts directed by [^18^F]-Fluoromisonidazole PET images had promising results, but failed to demonstrate significant advantage (28–30). This may be associated with inherent disadvantages of PET imaging, such as low contrast and limited spatial resolution, long hypoxia radiotracer uptake time (2–4 h), and limited temporal resolution of acute changes in hypoxia. Additionally, there is also no universal definition to define hypoxia based on radiotracer uptake, which may lead to different results across clinical sites and requires further research (31, 32).

The current study uses electron paramagnetic resonance oxygen imaging (EPROI) to locate and direct radiation boosts to local hypoxic tumor regions while minimizing radiation to oxygenated tumor regions and surrounding healthy tissue. EPROI is a non-invasive, quantitative tool for imaging pO_2_ *in vivo* (33). It uses a trityl spin probe (34) for pO_2_ quantification through the linear dependence of its electron spin–lattice relaxation rate on pO_2_. It measures pO_2_ to ~1 mmHg at low pO_2_, with spatial resolution ~1 mm, and, within statistical errors, no confounding variation, particularly self-relaxation (35). The lower-frequency 250-MHz EPR imager operation allows for a penetration depth accessible to human imaging. This low, penetrating radiofrequency argues direct translation of these preclinical oxygen imaging experiments into the clinic (36). Higher-frequency EPR imagers that generate higher signal-to-noise ratio – at the expense of penetration depth – were also used in this work.

Previous work showed EPROI images were spatially correlated with the measurements obtained with phosphorescence quenching (Oxford Optronics, Banbury, United Kingdom) (37). The OxyLite probe was stereotactically infused into the normal and tumor tissue of a mouse leg while in the imager resonator immediately after obtaining an EPROI. Exempting the artifact of superficial surgical scalpel entrance, the EPROI voxel measurements agree to within 2–3 torr comparing the fiberoptic probe launched into the highly heterogenous pO_2_ environments of mouse tumors from each of nine animal tumors (37). EPROI hypoxic fractions of 10 to 15% in murine sarcomas and mammary adenocarcinomas predicted clonogenic control with high significance when treated with a 50% control dose, supporting the value of the exacting paradigm (38). Local validation of EPROI in minimally metastatic tumor models requires a tumor control paradigm.

A crucial aspect of this study is its emphasis on the reduction of the probability that the last clonogen – the last malignant and most resistant cell that could survive – would proliferate into a more radioresistant and recurrent tumor. We refer to this paradigm as a clonogenic control experiment (39, 40). It stands in contrast to growth delay paradigms which are less expensive in many aspects, particularly in animal numbers, but emphasize response of the most sensitive cells and portions of a localized malignancy. The results from a previous clonogenic control study in preclinical FSa fibrosarcomas (41) is the first mammalian demonstration that selective hypoxic resistance targeting significantly improves tumor control.

Given the success in demonstrating enhanced tumor control in one mouse model, it is imperative to study tumor hypoxia across several tumor types, and its response to various radiation therapy regimens. Three tumor murine models were evaluated for oxygen image-guided radiation therapy: FSa fibrosarcomas, MCa-4 mammary adenocarcinomas, and SCC7 squamous cell carcinomas. These solid tumors have been previously used for imaging hypoxia (38, 42), and were chosen for their low rate of metastasis to adequately represent local control as the basis for clonogenic control. They include three major tumor types with variability in tumor growth rate, radiation control doses, and histopathology. For example, FSa tumors have a higher instance of necrosis and heterogenous tumor cell density; MCa-4 tumors contain large structures of vasculature, stroma, and collagen; SCC7 tumors have densely packed cells and dense microvasculature (43). These features result in, or are caused by, tumor hypoxia.

The primary objective of this study is to confirm the effects of hypoxic vs. oxygenated boosts on local tumor control probability (LTCP) in two other tumor types. The secondary objectives are to understand the effects of tumor volume, hypoxic fraction, and delay between imaging and different radiotherapy doses on LTCP.

## 2. Materials and methods

Tumors were used for imaging and treatment once grown to within specific volume limits (between 225–750 mm^3^ for FSa tumors determined by caliper measurements and 225–450 mm^3^ for MCa-4 and SCC7 tumors determined from T2 MRI in this work) and hypoxic fraction at 10 mmHg (HF10) between 0.02–0.42. HF10 was calculated by dividing the number of hypoxic tumor voxels defined from EPROI by the total number of tumor voxels defined from the T2 MRI. These limits were chosen to ensure animal comfort, while being large enough to develop hypoxia. Tumors with an HF10 > 0.42 prevented randomization to oxygenated boosts with equivalent integral radiation volume. Because HF10 for all 3 tumor types poorly correlated with tumor volume, a large number of animals had to be excluded from the study (Supplementary Table S1).

**Figure 1:**
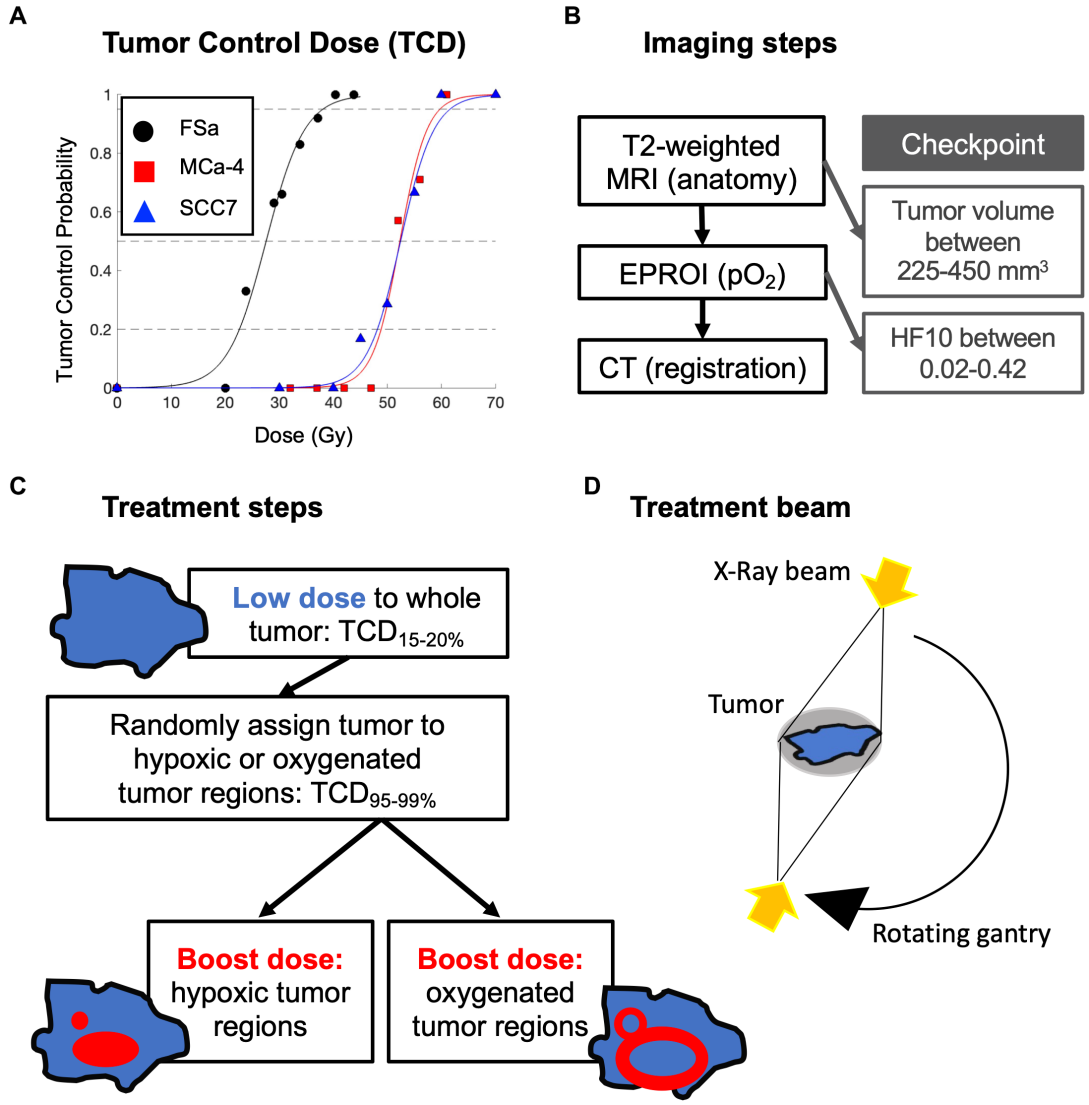
Overview of experimental design: **(A)** initial tumor control dose (TCD) studies to determine tumor type specific low whole tumor and boost doses (delivered sequentially) in a separate set of animals; **(B)** order of multimodal imaging and checkpoints to determine tumor eligibility for treatment; **(C)** whole tumor treatment followed by randomly assigned boost dose treatment with **(D)** opposing X-Ray treatment beams.

The full experimental design is summarized in Figure 1. Details of imaging and selection of hypoxia-target regions have been previously reported in Epel et al. (41), though critical points are recapitulated below.

### 2.1. Tissues and cell cultures

Syngeneic FSa fibrosarcoma, MCa-4 mammary adenocarcinoma, and SCC7 squamous cell carcinoma tumor cells were obtained from the M.D. Anderson Cancer Center. Frozen 8th generation FSa and 4th generation MCa-4 tumor material was grown in the flanks of C3H mice and harvested. The SCC7 frozen cell material was expanded *in vitro* and harvested. Harvested 0.1–4.0 × 10^6^ cells were suspended in modified Eagle medium with 10% fetal bovine serum and injected in the gastrocnemius muscle of the left leg of the mice. Tumor growth rates varied by tumor type: 120 mm^3^/day for FSa, 54 mm^3^/day for MCa-4, and 68 mm^3^/day for SCC7 lines.

### 2.2. Animal model, anesthesia, and euthanasia

Animal experiments followed US Public Health Service policy, NIH Guide for the Care and Use of Laboratory Animals, and were approved by the Institutional Animal Care and Use Committee. Mice were observed three times per week and were euthanized and removed from the study if they exhibited signs of infection, injury, or tumor regrowth to larger twice the volume of the tumor at treatment to minimize pain and suffering. Euthanasia was performed with isoflurane overdose or CO_2_ asphyxiation, confirmed by cervical dislocation.

Female C3H/HeNCrl tumor-bearing mice were used in all experiments. At the time of imaging and treatment assignment, the mice were ~ 12 weeks old. To prepare the mice for imaging, anesthesia was induced using 2% isoflurane mixed with air (21.5% oxygen and 78.5% nitrogen) and maintained with 1.5% isoflurane and air, administered with a mask. Respiration rate was maintained at approximately 1.5Hz, which was maintained by varying the isoflurane concentration, and core temperature was kept at 37°C monitored continuously with a SA Instruments system (Stony Brook, NY).

### 2.3. Imaging

To prepare the mouse for imaging, the tumor-bearing leg was set in a soft rubber half-circumferential vinyl polysiloxane dental mold cast (GC America) which fit into a 3D printed plastic support bed to ensure consistent immobilization between imaging modalities. The tail vein was cannulated to deliver the EPROI pO_2_ quantifying spin probe IV.

**Figure 2:**
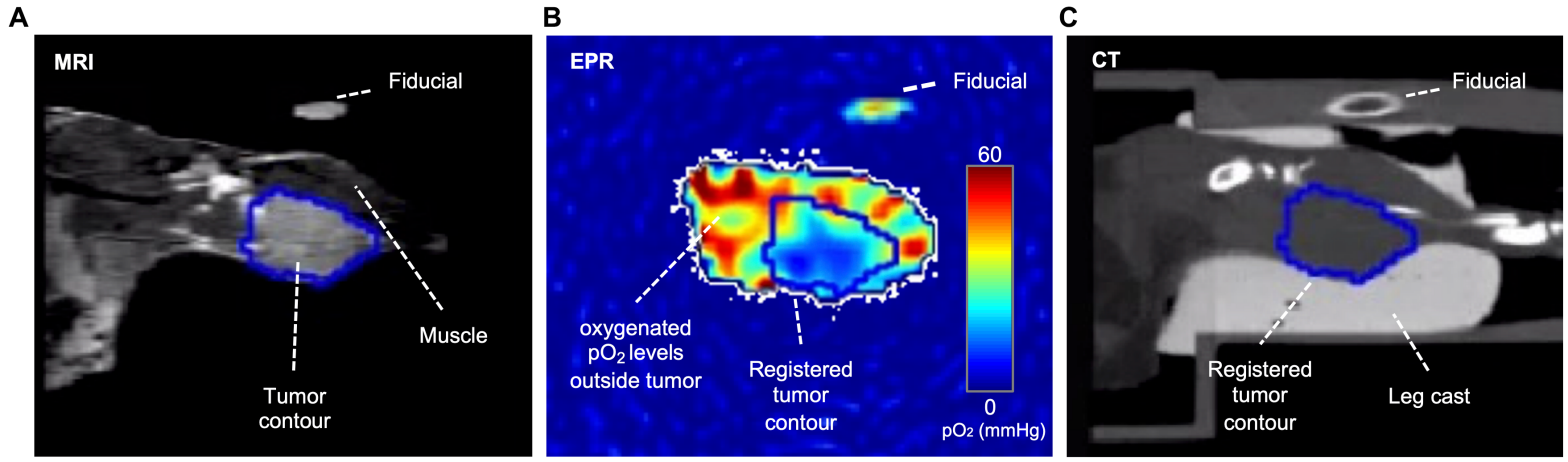
Coronal plane of an example tumor slice in three modalities. In the T2-weighted MRI **(A)**, voxels within the tumor are brighter than in the muscle; therefore, the tumor contour was based on T2-weighted MRI images, which were then registered to CT and EPR via fiducials. In the EPR image **(B)**, the tumor is often more hypoxic than surrounding healthy tissue, though hypoxia is heterogeneously distributed. The CT **(C)** shows the cast and bed, which are essential for registration and dose planning. A fiducial filled with water and oxygen spin probe appears in all three modalities.

For radiation treatment planning, three types of images were obtained: T2-weighted MRI (Figure 2A) to define tumor boundary, EPROI to define hypoxic region boundaries (Figure 2B), and CT (Figure 2C) to register all to the location of the X-RAD 225Cx (Precision X-Ray, North Branford, CT, United States) radiation source, which is also the source of the cone-beam CT. Fiducials filled with water and trityl spin probe were embedded in the imaging bed to guide image registration between modalities.

T2-weighted MRI is the primary mode to localize and target curative radiation therapy (44). A 9.4 Tesla small animal scanner (Bruker, Billerica, MA, United States) was used with a multislice RARE sequence yielding 0.1 × 0.1 × 0.75 mm^3^ voxel resolution. If the MRI determined tumor volume was within range, the mouse was selected and awakened for further imaging and treatment.

EPR pO_2_ images were acquired with pulse spin–lattice relaxation oxygen imaging using OX071 oxygen sensitive spin probe infused IV and a low-frequency 250MHz EPR imager (35, 45) or a higher-frequency 720MHz EPR imager (JIVA-25^™^, O2M, Chicago, IL). The pO_2_ was imaged using 135 μL of 72mM OX071 (0.43 mmols/Kg) whose spin lattice relaxation was a remarkably linear function of pO_2_ (35, 45). OX071 is a triacid with extracellular distribution (24). The probe has a maximum tolerated dose of 2.5–7 mmol/kg (46); the maximum dose used in this study was 0.45–0.9 mmols/kg. OX071 is synthesized by the Novosibirsk Institute of Organic Chemistry (47) and also available from GE Healthcare (Little Chalfont, Buckinghamshire, United Kingdom).

Fiducials were first imaged in EPROI for registration purposes, followed by three pO_2_ images. The spin probe was administered with an initial bolus followed by 3.5 μL/min continuous infusion. Each EPR pO_2_ image acquisition took 11 min with the 250-MHz EPROI system (total 33 min), and 5 min with the JIVA-25^™^ system (total 15min). The second of three pO_2_ images with isotropic 0.67mm voxel resolution was used to assess the location of all hypoxic voxels (pO_2_ ≤ 10mmHg) and calculate HF10.

An X-RAD 225Cx provided CT images of the tumor bearing leg to locate the radiation target volume and deliver 2 Gy/min to both whole tumor and boost radiation doses.

### 2.4. Determining dose and treatment

Prior tumor control dose (TCD) studies were used to determine the whole tumor-specific low dose that would result in 15–20% LTCP, and high dose that would result in 95–99% LTCP. An initial TCD finding study was completed on 48 FSa, 47 MCa-4, and 50 SCC7 tumors (Figure 1A). A group of tumors of each type was treated with a range of doses with the X-RAD 225Cx irrespective of hypoxic fraction. Doses ranged from 20 to 45Gy for FSa tumors, 32 to 61Gy for MCa-4 tumors, and 30 to 70Gy for SCC7 tumors providing dose vs. control curves. Based on those TCD curves, TCD_15-20%_ whole tumor treatment and TCD_95-99%_ boost dose treatment were determined for each tumor type (Table 1). Whole tumor low-dose and boost treatments were delivered sequentially.

**Table 1:** Treatment doses, number of mice in each treatment group and overall, and median values of potential confounding variables for each tumor type.

	FSa fibro-sarcomas	MCa-4 mammary adenocarcinomas	SCC7 squamous cell carcinomas	SCC7 squamous cell carcinomas
EPROI operating frequency	250-MHz	250-MHz	250-MHz	720-MHz
Whole tumor dose (TCD_15-20%_)	22.5 Gy	49.9 Gy	48 Gy	48 Gy
Total boost dose (TCD_95-99%_)	35.5 Gy	62.9 Gy	61 Gy	61 Gy
Hypoxic boost (N)	29	26	23	21
Oxygenated boost (N)	25	22	21	17
Total (N)	54	48	44	38
Tumor volume (mm^3^)	391	342	349	356
Hypoxic fraction	0.14	0.14	0.22	0.15
Treatment delay (hours)	1.2	3.5	2.9	2.8

**Figure 3:**
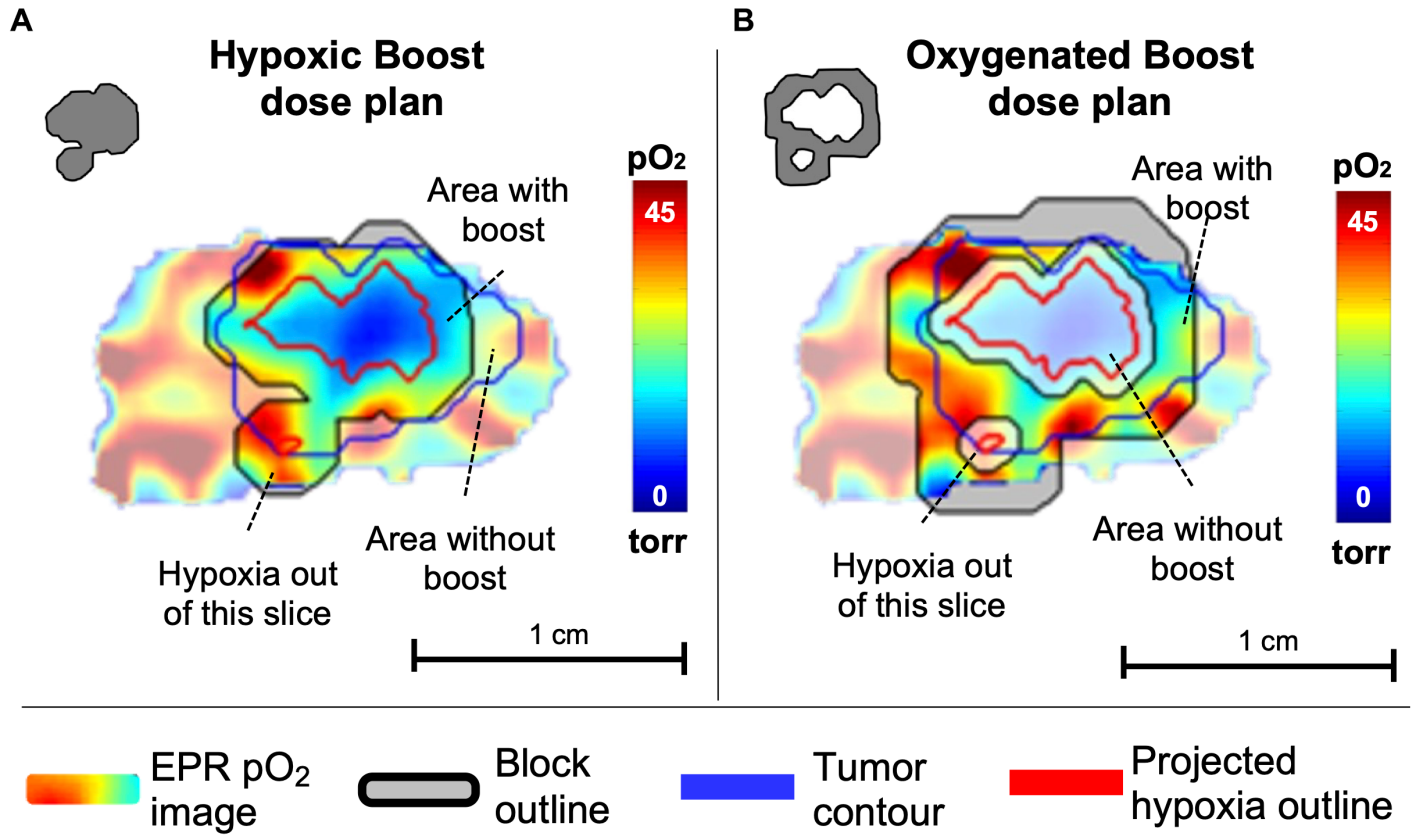
Example of a central slice of a tumor, quantitatively displaying pO_2_ levels in which pO_2_ ≤ 10 torr (=mmHg) are considered hypoxic. pO_2_ contours represent projections of 10 torr boundaries of all tumor hypoxia onto the central slice. **(A)** The tumor was randomly assigned to hypoxic boost treatment, in which only the hypoxic region (grey in the upper left insert) received a boosted TCD_99%_ dose. **(B)** The tumor was randomly assigned to oxygenated boost treatment (grey in the upper left insert), where the hypoxic region is blocked off but a similar integral dose is deposited in the tumor. Note that the projected hypoxia outlines in red include regions outside the displayed plane. Margins for boost blocks included 1.2 mm for Hypoxia Boost and 0.6mm for Oxygenated Boost.

For treatment, each mouse was randomly assigned to either a Hypoxic Boost or Oxygenated Boost treatment. The High-Risk planning target volume (PTV_HR_), the hypoxic boost region, was defined by the EPR pO_2_ image within the MRI-based tumor contour projected onto the central slice, with a 1.2mm margin added to the hypoxic region (Figure 3A). The Low-Risk planning target volume (PTV_LR_), the oxygenated boost region, was planned similarly, with a 0.6mm margin around the hypoxic regions defining the inner edge of the boost region, and the outer edge was expanded to an approximately equal aperture area to that of that tumor’s hypothetical hypoxic boost (Figure 3B), centered around the beam. The 0.6mm margin was created to avoid any boost dose spillage over the hypoxic subvolume due to the scattering phenomenon or any setup uncertainty. Expanding the PTV_LR_ treatment area to the same aperture area as the PTV_HR_ was to ensure that differences in treatment outcome were not caused by a difference in integral dose between Hypoxic and Oxygenated Boost treatments.

Delivered dose was validated by Gafchromic EBT3 medical dosimetry film (Ashland, Fiskeville, RI) calibrated with an ion chamber. A more detailed explanation of the selection of hypoxia-target regions, radiation block fabrication, and radiation boost region determination is in previously published work (41).

### 2.5. Monitoring tumor control

After successful boost treatments, mice were recovered from anesthesia. MCa-4 and SCC7 tumors were observed for up to 180 days for local tumor recurrence. FSa tumors were observed for up to 90 days. Tumors were defined as “locally recurrent” when caliper measurements exceeded twice the tumor volume as the day of treatment, “controlled” when there was no local recurrence by 180 days for MCa-4 and SCC7 tumors (or 90 days for FSa tumors), or “cencored” if the animal had to be euthanized for reasons other than local tumor recurrence. When necessary, time of recurrence was interpolated between measurements to the estimated day.

### 2.6. Statistical methods

Two-sample *t*-tests were conducted to test for significant differences between means of risk factors for local recurrence between the two treatment groups (Hypoxic vs. Oxygenated Boost) before radiotherapy. Risk factors and potential confounders include tumor volume, HF10, and delay between imaging and treatment.

Progression-free (local tumor control) survival was compared between the Hypoxic Boost and Oxygenated Boost treatments using Kaplan–Meier survival analysis (48). Significant differences were determined using the two-sided log-rank test. Cox regression models were used to estimate the hazard (risk) ratio of local recurrence for Hypoxic vs. Oxygenated Boost treatments, adjusting for the aforementioned potential confounding variables. The proportional hazards assumption was verified using the global Schoenfeld test.

Additional Kaplan–Meier survival analysis used the stratified log-rank test to group tumors by potential confounding variables: HF10 with low/high hypoxic fraction, high/low tumor volume groups, and short/long delay between imaging and treatment. These classifications were relative to the median value of each tumor type (Table 1).

Data analysis was conducted using the statistical software R. R markdown files can be obtained from the authors upon request.

## 3. Results

With the 250-MHz EPR imager, a total of 78 mice were entered in the FSa group, 65 mice in the MCa-4 group, and 70 mice in the SCC7 group. With the 720-MHz EPR imager, 48 mice were entered in the SCC7 study. Following exclusion criteria for tumors out of range of pre-determined hypoxic fractions, tumor volumes, and experimental failures in the process of radiation, the following total numbers of mice were included in statistical analysis: N = 54 for FSa, N = 48 for MCa-4, N = 82 for SCC7 tumors (N = 44 with the 250-MHz, and N = 38 with the 720-MHz EPR imagers). Supplementary Table S1 summarizes the total number of animals entered in each tumor histology’s study, and reasons for exclusion.

**Figure 4:**
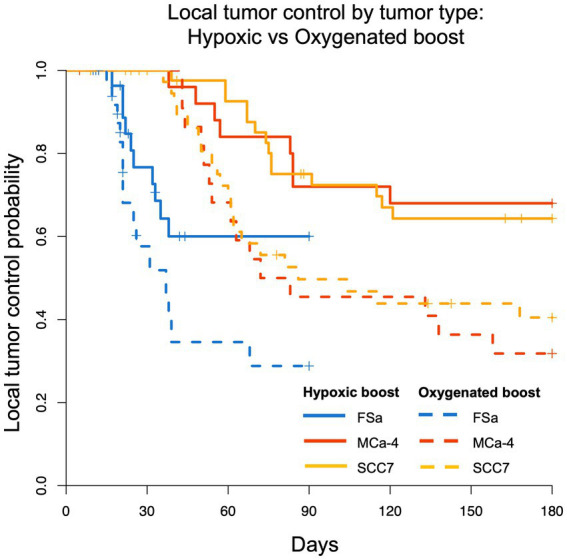
Kaplan–Meier LTCP survival comparing Hypoxic Boost (solid line) with Oxygenated Boost (dashed line) for FSa (blue), MCa-4 (red), and SCC7 (yellow) tumor types. For all tumor types combined, LTCP is significantly different with Hypoxic Boost (*p* < 0.001) compared to Oxygenated Boost treatments using the log-rank test.

Figure 4 shows the Kaplan–Meier estimate of LTCP curves for each tumor type (FSa in blue, MCa-4 in red, and SCC7 in yellow) grouped by Hypoxic Boost (solid line) vs. Oxygenated Boost (dashed line). Using the stratified (by tumor type) log-rank test to test for differences in the LTCP curves by boost treatments, we found that there was a significant advantage in LTCP for tumors treated with Hypoxic vs. Oxygenated Boost (*p* < 0.001). Using the log-rank test for each tumor type, the significance of Hypoxic Boost advantage for LTCP was reached at *p*-values of *p* = 0.04 for FSa, *p* = 0.01 for MCa-4, and *p* = 0.02 for SCC7 tumors.

Using Cox regression analysis for all tumor types, the hazard ratio (HR) was below 0.5 for the risk of recurrence when treating tumors with Hypoxic Boost relative to Oxygenated Boost, adjusting for tumor volume, HF10, and treatment delay. Specifically, for FSa tumors, HR = 0.21 (*p* = 0.02); for MCa-4 tumors, HR = 0.36 (*p* = 0.065); for all SCC7 tumors, HR = 0.44 (*p* = 0.02). These results showed that Hypoxic Boost has a smaller risk of recurrence than Oxygenated Boost adjusting for the effects of potential confounders, and were consistent with our observations from Kaplan–Meier analysis. Table 2 summarizes the HR and significance of each tumor type and potential confounding variable. Across all tumor groups, we did not find a significant effect of delay between imaging and treatment on tumor time-to-recurrence. This is an important observation for the concern about the effects of cyclical or changing hypoxia over time between imaging and treatment. The effect of tumor volume or HF10 on mice treated with Hypoxic Boost vs. Oxygenated Boost was dependent on tumor type.

**Table 2:** Hazard ratios for time-to-recurrence of Hypoxic Boost vs. Oxygenated Boost treatments based on Cox regression adjusted for potential confounders: tumor volume, HF10, and delay between imaging and treatment.

Tumor group	FSa (250-MHz)	MCa-4 (250-MHz)	SCC7 (all)	SCC7 (250-MHz)	SCC7 (720-MHz)
Hypoxic boost	0.21*	0.36*	0.44 *	0.81	0.20 **
Tumor volume	0.87	0.91	1.5	1.2	2.2 *
Delay	0.9	0.98	1.2	1.1	0.98
HF10: Hypoxic boost	0.27 **	0.53	1.4	2.4	0.49 *
HF10: Oxygenated boost	1.1	0.27 **	1.0	1.2	1.5

Values of *p* ≤ 0.05 are marked with * and values of *p* ≤ 0.01 are marked with **.

Supplementary Figure S1 shows the distribution of tumor volume, HF10, and treatment delay across tumor types for Hypoxic vs. Oxygenated Boost treatments. The two-sample *t*-test did not show a significant difference between treatment groups with one exception (treatment delay for MCa-4 tumors). However, Cox regression analysis shows that treatment duration did not affect the results. Pearson correlation analysis to assess the linear correlation between HF10 and tumor volume did not show a linear relationship between the two tumor features (Supplementary Figure S2), highlighting the importance of using oxygen imaging to infer the extent of tumor hypoxia.

Kaplan–Meier estimates of LTCP curves stratified by low/high tumor volume, low/high HF10, and short/long treatment delay for all tumors are shown in Supplementary Figure S3. Stratified curves for SCC7 tumors grouped by EPR imager are shown in Supplementary Figure S4. A key observation for SCC7 tumors from the 720-MHz EPR imager is that tumors with low HF10 treated with Hypoxic Boost have a 180-day LTCP of 0.9, while tumors with high HF10 have a 180-day LTCP of 0.4. This is the opposite effect of what is observed with FSa tumors, again highlighting the importance of performing these experiments in different tumor types and with a wide range of HF10.

When grouping SCC7 tumors by EPR imager, those imaged with 720-MHz EPROI showed a significantly improved LTCP with a Hypoxic Boost (*p* = 0.007). However, we did not identify a significant difference between the boost treatment groups for SCC7 tumors imaged with 250-MHz EPROI (*p* = 0.4). This was likely due to SCC7 tumors from the 250-MHz EPROI group having >50% higher HF10 than all other groups (Supplementary Figure S1E). This again shows that HF10 plays a different, significant role in treatment outcome in SCC7 tumors.

## 4. Discussion

This work presents data in three preclinical mammalian tumor types to demonstrate that accurate targeting of hypoxic tumor subvolumes with a boost of radiation improves the LTCP relative to a boost of the same integral dose to oxygenated regions of the tumor. This provides additional biologic validation of EPROI in targeting enough resistant hypoxic tumor subvolumes to increase the probability of eliminating all clonogens. It may provide a means of reducing the dose to oxygenated regions and delivering a radiation boost to resistant hypoxic regions. This can reduce dose to potential organs at risk that are related to quality of life, and still improve tumor control. It promises the enhancement of the therapeutic ratio.

A notable difference in the study design of this preclinical work, compared to clinical dose escalation studies, is the low dose delivered to oxygenated tumor regions. In this preclinical study, a lower 15–20% TCD was delivered to the whole tumor followed by a boost to reach TCD >98%. In clinical studies using [^18^F]FMISO PET to locate hypoxia, a high dose was delivered to a whole tumor followed by a more cautious boost dose. For example, in Vera et al., 66 Gy was delivered to the whole tumor for non-small cell lung carcinomas, followed by a 4–13 Gy boost dose (depending on the tumor site and organs at risk) (29). In Welz et al., 70 Gy was delivered to the whole tumor for head and neck cancers, followed by a 7 Gy boost (30). The results we present in this work are promising for dose reduction, even when delivering a significantly lower dose to the oxygenated tumor in one fraction.

Clinical efforts with spatially fractionated radiation therapy (LATTICE) have also been achieved with promising results (19, 49–52). Ferini et al. conceptualized and clinically tested the usefulness of a non-homogeneous irradiation based on the heterogeneity of tumor metabolism (as detected in [^18^F]FDG PET) or functionality [as evidenced by the apparent diffusion coefficient (ADC) sequence in MRI] as a mirror of a varying oxygenation across the tumor volume. While being aware of the inability of [^18^F]FDG PET to discriminate between the well- and poorly-oxygenated tumor areas (due to the Warburg and Pasteur effects), they assumed that boost doses in tumor areas with changes in SUV may increase the chance of hitting some hypoxic clones (49, 50). Similarly, the ADC map may reflect the cellular density as a surrogate of oxygen supply of a proliferating tissue (51). ADC MRI has also been investigated by other groups as an indirect method of non-invasive imaging markers of hypoxia (53, 54).

The aforementioned authors, though not irradiating the whole suspected hypoxic tumor compartment, achieved excellent clinical results, likely stemming from an efficient recruitment of the local host immune response. The partial irradiation of hypoxic tumor subvolumes could further reduce the radiation dose to OARs to improve therapeutic index. Finally, a LATTICE approach guided by the tumor oxygenation landscape could represent an optimal and sufficient bridge between the two techniques (19, 49).

Interestingly, the SCC7 (720-MHz) group showed a significant difference between Hypoxic and Oxygenated Boost treatments (*p* = 0.007) while the SCC7 (250-MHz EPROI) group failed to show a significant difference between treatment groups (*p* = 0.4). However, the 250-MHz group had over 50% higher HF10 compared to all other groups, with median HF10 = 0.22 compared to median HF10 for other tumor groups that ranged from 0.14–0.16 (Supplementary Figure S1D). However, within each tumor type between the two treatment plans, the distribution of HF10 and tumor volumes were comparable, showing that there was no confounding variation caused by the risk factors discussed for tumor recurrence. The dependence on hypoxic fraction in SCC7 tumors of the curative advantage from targeting hypoxia may be an example of a crucial addition to personalized treatment provided by an accurate pO_2_ image. It can provide guidance determining where optimum treatment requires a more aggressive approach with higher boost doses and possible increased risk of OAR side effects. Or it may suggest that radiation should be combined with other treatment modalities.

The impact of low vs. high HF10 also varied across tumor types, which shows itself as a critical biomarker in radiation dose painting experiments. Hypoxic Boost treatments with high HF10 showed a higher LTCP for both FSa and MCa-4 groups. Hypoxic Boost treatments with low HF10 showed a higher LTCP for both SCC7 groups. This implies a tumor type-dependent effect of HF10 on treatment outcome, where low HF10 increases LTCP for SCC7. All tumor types in this study have intact immune systems. High levels of hypoxia are well known to interfere with tumor immunogenicity (55). This may indicate that SCC7 tumors require higher boost doses to hypoxic tumor subvolumes compared to FSa and MCa-4 tumors.

The 1.2mm margin was added to the PTV_HR_ in the presented work to ensure that at least 98% of hypoxic tumor regions were exposed to a boost of radiation, and to account for minor positioning or image registration errors. Even small, disconnected spatters of hypoxic regions were targeted to radiate as many clonogenic cells as possible. However, the margin increased the fraction of radiation to oxygenated tumor. The distribution of hypoxic vs. oxygenated voxels in the beam irradiated for both treatments is shown in Supplementary Figures S5–S6. This highlights a limitation to the design of opposed radiation beam treatments, where expanding the PTV_LR_ treatment area to the same aperture area as the PTV_HR_ was centered around the beam rather than expanded into the tumor. This method resulted in a fraction of the boosted beam delivered outside the tumor, either into healthy tissue in the leg or avoiding the leg altogether. This was most apparent in SCC7 tumors imaged in the 250 MHz EPR imager, a group with higher HF10 values, therefore leaving fewer oxygenated voxels to treat.

The design of the radiation treatments at once provided the cleanest separation between boosts to hypoxic and oxygenated tumor, using opposed fields with margins to minimize inclusion of the voxels of unwanted identity. This, however, left out voxels shadowed by those of unwanted identity and limited the extent of hypoxia allowable for randomization. Relative to modern IMRT, which can provide more subtle sculpting of dose distributions with capability to define 3D avoidance structures and more carefully defined “simultaneous integrated boosts,” these approaches are more primitive. A hypothesis for the lower LTCP for SCC7 tumors from the 250-MHz EPROI group, other than a higher HF10, is the higher percentage of voxels outside the tumor “treated” with a boost dose (see Supplementary Figure S6A). Work involving compensators to address this limitation and provide the doses sculpting is ongoing in the laboratory (56).

Another limitation of the study was the relatively high number of mice inoculated with tumor cells whose tumor sizes fell out of the study’s radiobiological range by the scheduled experiment date (see Supplementary Table S1). The range of tumor growth rates led to some tumors being too large to be included in the study, which were sacrificed.

## 5. Conclusion

This work demonstrates that tumor hypoxia boosts defined by locally validated EPR pO_2_ images in three preclinical tumor types provided at least a factor of ~2 enhancement in tumor control relative to oxygenated tumor boosts of similar integral dose. This is evidence for mammalian hypoxia as a target for increased enhanced radiation dose relative to that for oxygenated tumor. The clonogenic survival assay used here is crucial for the study of the most resistant tumor cells, despite its increased complexity. The LTCP for the oxygenated tumor boost animals was improved relative to the control for animals treated to the whole tumor dose in the TCD studies, but only by a much smaller fraction, ~10%. Clearly there are other sources of tumor resistance indicated by the incomplete LTCP from the hypoxic boosts and the small improvement in the oxygenated tumor LTCP. Ongoing work involves a more sophisticated dose planning for small animal IMRT to better emulate treatments more relevant to clinical treatment design (32, 56).

## Data availability statement

The original contributions presented in the study are included in the article/Supplementary material. Further inquiries can be directed to the corresponding author.

## Ethics statement

The animal study was approved by the University of Chicago Institutional Animal Care and Use Committee. The study was conducted in accordance with the local legislation and institutional requirements.

## Author contributions

IG: Formal analysis, Writing – original draft, Writing – review & editing. BE: Conceptualization, Data curation, Formal analysis, Methodology, Resources, Software, Writing – review & editing. MiG: Formal analysis, Resources, Software, Writing – review & editing. EB: Data curation, Resources, Writing – review & editing. JL: Data curation, Resources, Writing – review & editing. KH: Data curation, Resources, Writing – review & editing. JM: Data curation, Resources, Writing – review & editing. MeG: Data curation, Resources, Writing – review & editing. MM: Data curation, Resources, Writing – review & editing. RM: Data curation, Resources, Writing – review & editing. SS: Resources, Writing – review & editing. MK-S: Data curation, Methodology, Writing – review & editing. EP: Resources, Writing – review & editing. BA: Resources, Writing – review & editing. RW: Conceptualization, Writing – review & editing. VT: Resources, Writing – review & editing. MK: Conceptualization, Methodology, Writing – review & editing. HH: Conceptualization, Methodology, Supervision, Writing – review & editing.
