# Corrigendum: Absolute oxygen-guided radiation therapy improves tumor control in three preclinical tumor models

**DOI:** 10.3389/fmed.2023.1339872

**Published:** 2023-12-05

**Authors:** Inna Gertsenshteyn, Boris Epel, Mihai Giurcanu, Eugene Barth, John Lukens, Kayla Hall, Jenipher Flores Martinez, Mellissa Grana, Matthew Maggio, Richard C. Miller, Subramanian V. Sundramoorthy, Martyna Krzykawska-Serda, Erik Pearson, Bulent Aydogan, Ralph R. Weichselbaum, Victor M. Tormyshev, Mrignayani Kotecha, Howard J. Halpern

**Affiliations:** ^1^Department of Radiation and Cellular Oncology, The University of Chicago, Chicago, IL, United States; ^2^Department of Radiology, The University of Chicago, Chicago, IL, United States; ^3^Center for EPR Imaging in vivo Physiology, The University of Chicago, Chicago, IL, United States; ^4^O2M Technologies, Chicago, IL, United States; ^5^Department of Public Health Sciences, The University of Chicago, Chicago, IL, United States; ^6^Department of Biophysics and Cancer Biology, Jagiellonian University, Kraków, Poland; ^7^Novosibirsk Institute of Organic Chemistry, Novosibirsk, Russia

**Keywords:** hypoxia, oxygen, electron paramagnetic resonance, preclinical imaging, radiotherapy

In the published article, there was an error in the Funding statement. The National Institutes of Health grant R01 CA236385 was missing in the statement. The correct funding statement appears below.

