# Supplementary material for: Absolute oxygen-guided radiation therapy improves tumor control in three preclinical tumor models
Source: Front Med (Lausanne). 2023 Oct 12;10:1269689. doi: 10.3389/fmed.2023.1269689 (PMC10613495; doi:10.3389/fmed.2023.1269689)
Supplement: Supplementary file 1 [file Presentation_1.pdf]

Supplementary Material

1 Supplementary Figures

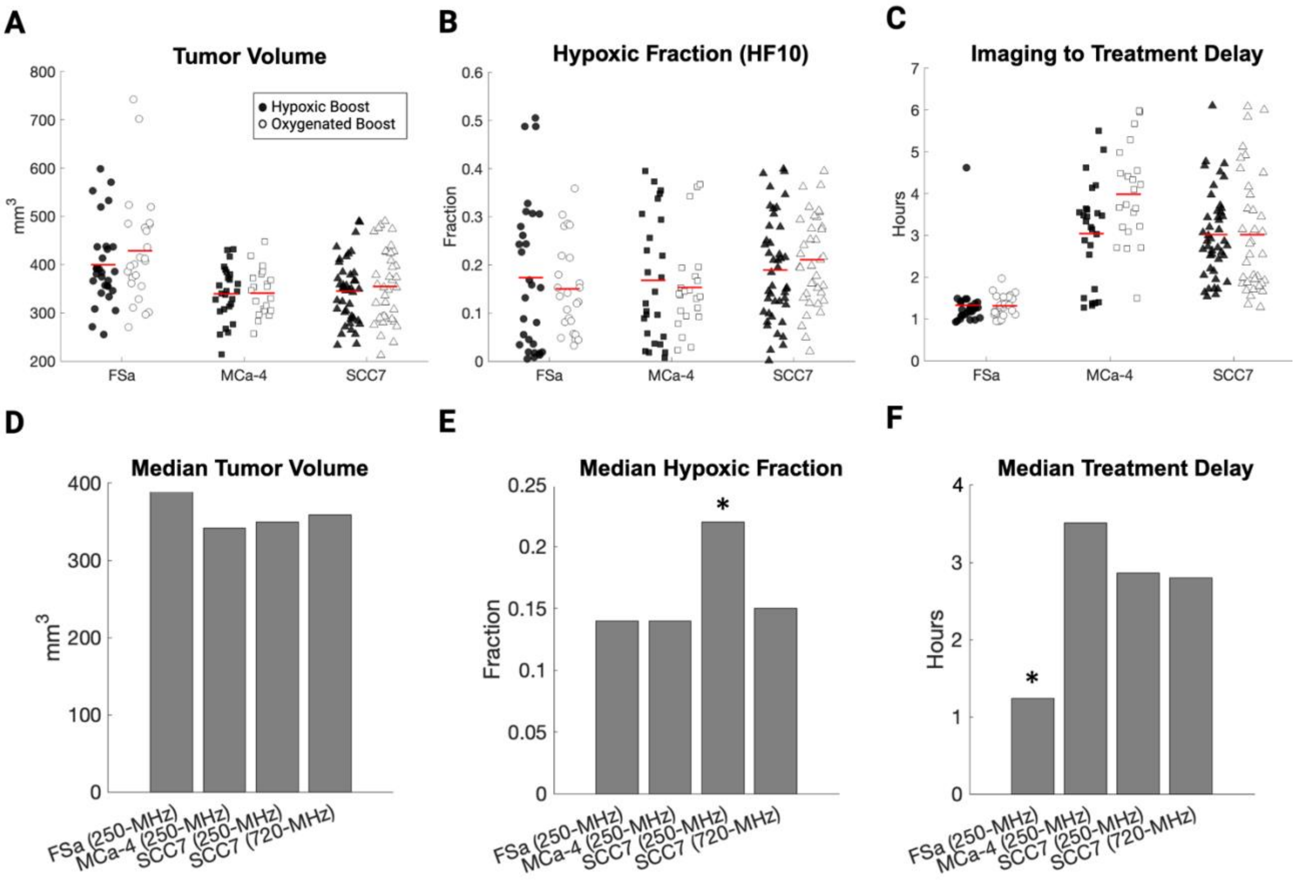

Supplementary Figure S1: Vertical dot plots showing the distribution of (A) tumor volumes, (B) hypoxic fractions, and (C) delay between imaging to treatment for each tumor type for mice treated with Hypoxic Boost (black) and Oxygenated Boost (white). Red bars show mean values for each tumor type and treatment group. Two-sample t-tests showed a significant difference in mean delay between the imaging and treatment delivery for MCa-4 tumors ($p<0.05$). Barplots D-F show median values for each tumor type, including the two EPR scanner types for SCC7 tumors (250-MHz vs 750-MHz). The SCC7 tumors whose pO_2 images were obtained at 250 MHz had a significantly higher median HF10 indicated by the * of greater than 50% ($p<0.05$).

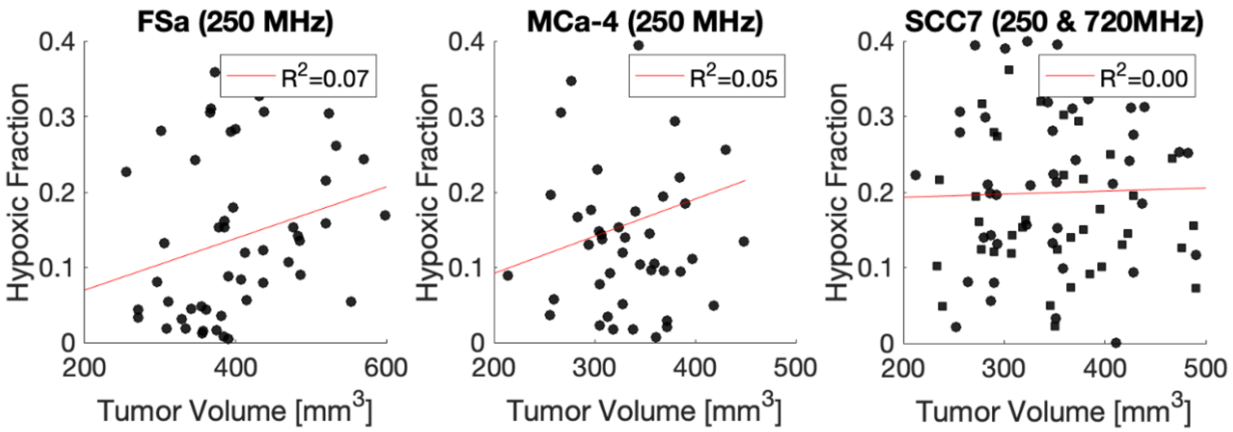

Supplementary Figure S2: Scatterplots of HF10 vs tumor volume for (A) FSa, (B) MCa-4, and (C) SCC7 tumors. Note that the variation in tumor volume explain only a small portion of the variation of HF10 evidenced by the low R^2 values obtained from the linear regression fits.

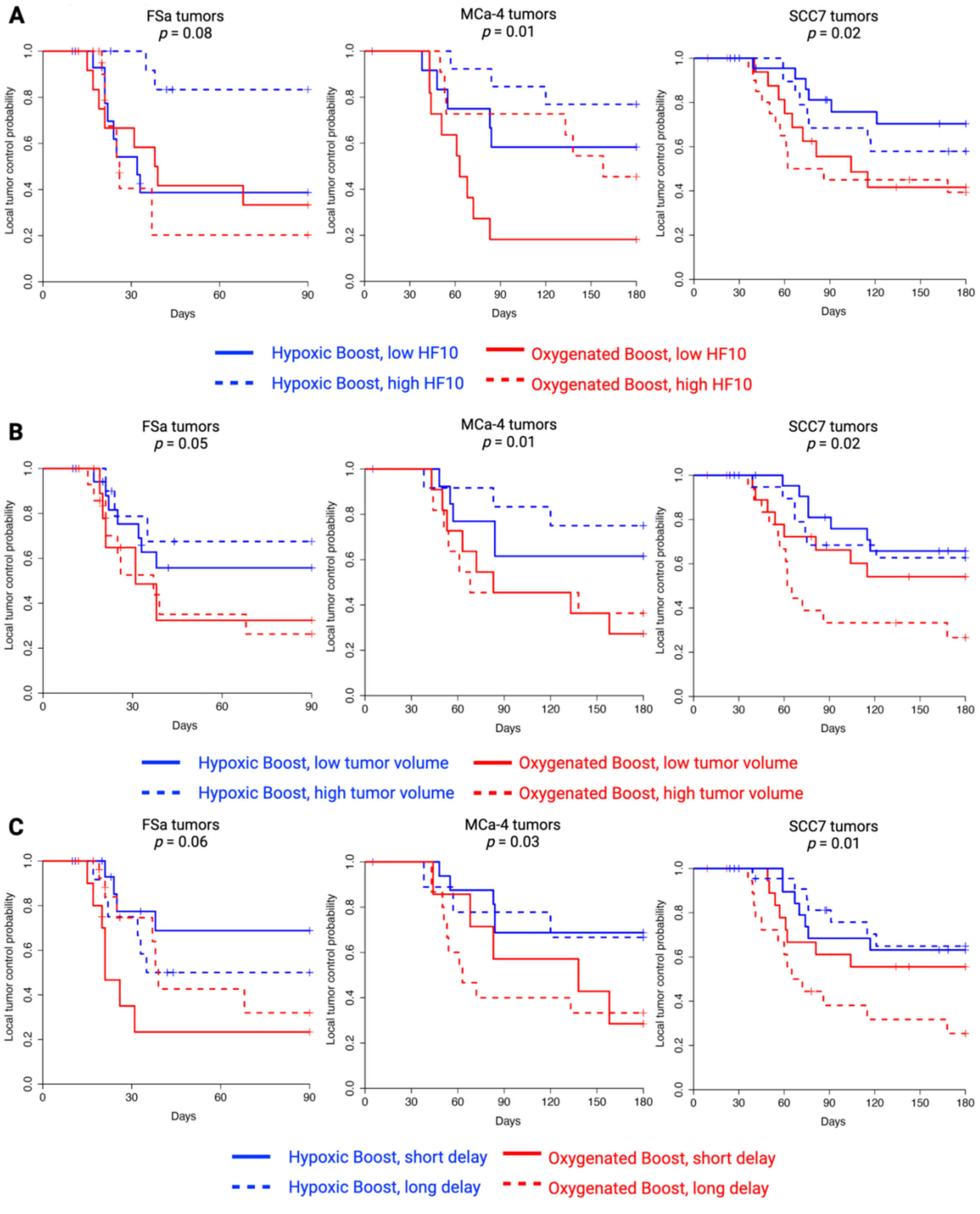

Supplementary Figure S3: Kaplan-Meier estimates of local tumor control probability curves for FSa, MCa-4, and SCC7 (250Mhz and 750MHz combined) tumors stratified by (A) low/high HF10, (B) low/high tumor volume, and (C) short/long delay between EPROI and radiation treatment. P-values calculated by stratified log-rank test.

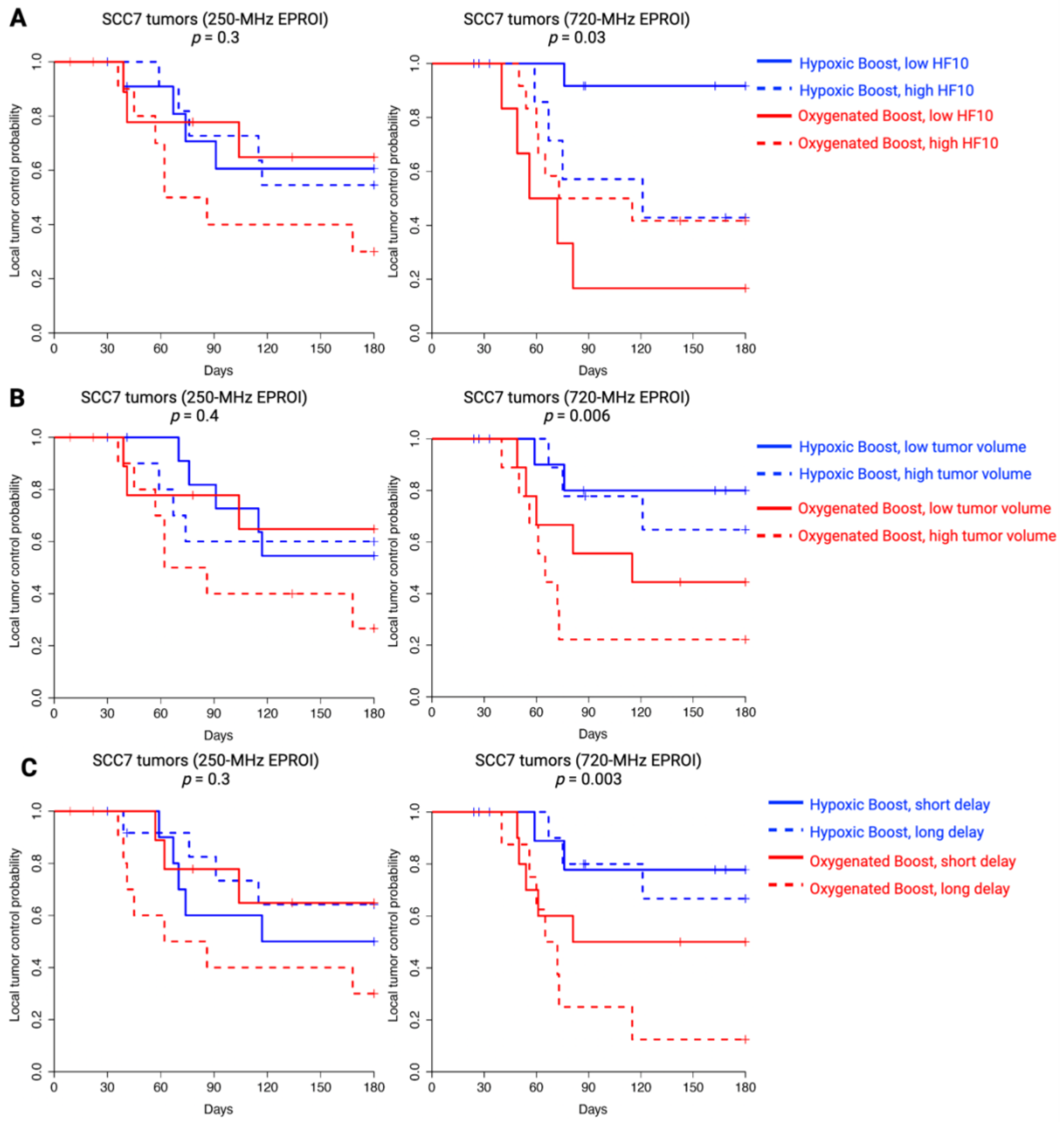

Supplementary Figure S4: Kaplan-Meier estimates of local tumor control probability curves for SCC7 tumors grouped by type of EPR imager – 250-MHz EPROI (n=44) and 720-MHz EPROI (n=38) – stratified by (A) low/high HF10, (B) low/high tumor volume, and (C) short/long delay between EPROI and radiation treatment. P-values calculated by stratified log-rank test.

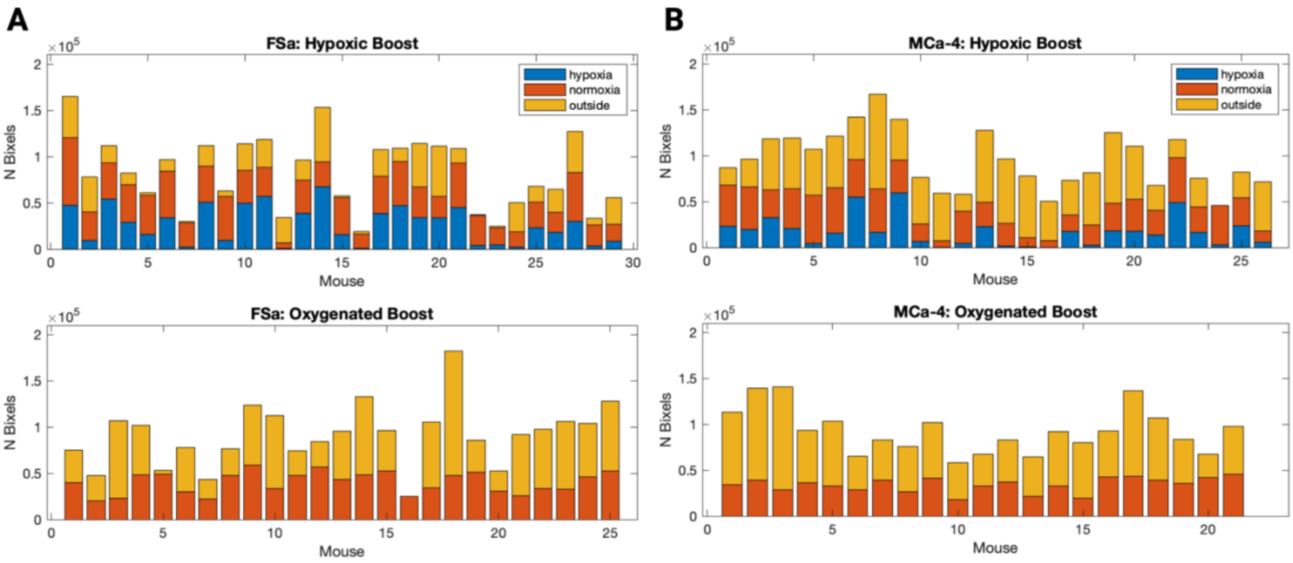

Supplementary Figure S5: Number of bixels treated with Hypoxic Boost (top) or Oxygenated Boost (bottom) for (A) FSa (A) and (B) MCa-4 tumor groups. The number of bixels that are hypoxic (blue), normoxic (orange), or outside the tumor (yellow) are stacked for each tumor.

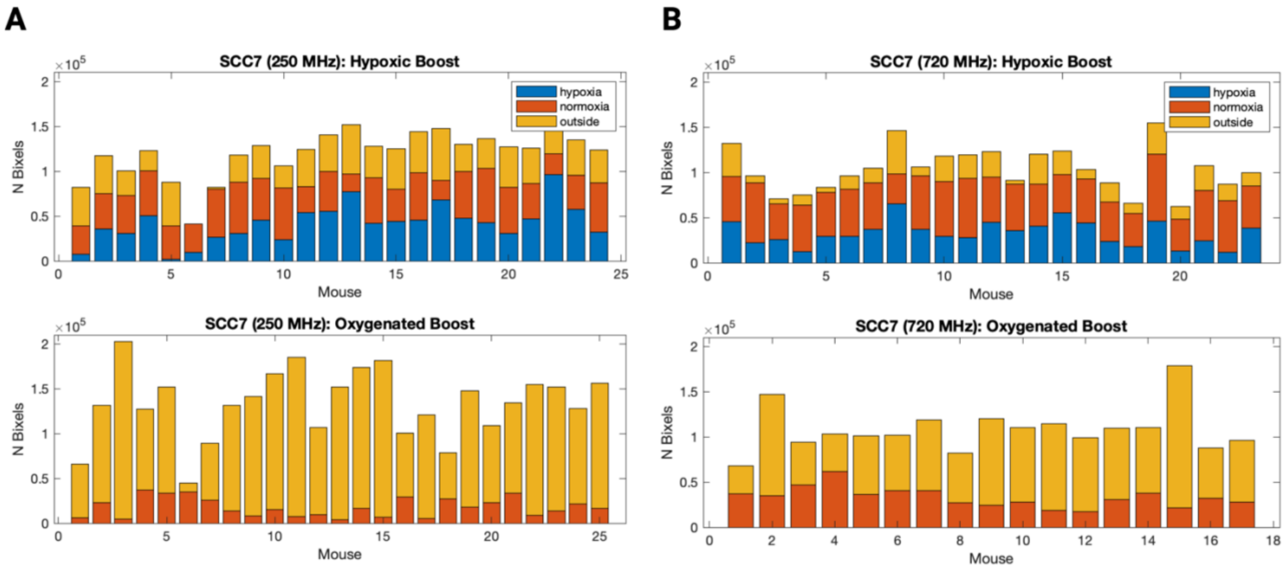

Supplementary Figure S6: Number of bixels treated with Hypoxic (top) or Oxygenated (bottom) Boost for SCC7 tumors imaged with the (A) 250-MHz and (B) 720-MHz EPR imagers. The number of bixels that are hypoxic (blue), normoxic (orange), or outside the tumor (yellow) are stacked for each tumor.

2 Supplementary Tables

Supplementary Table S1: Detailed reasons for animal inclusion/exclusion criteria.

Tumor histology	EPR imager	Animals included (N)	Animals excluded (N) due to:	Animals included in statistical analysis (n)
FSa fibrosarcomas	LF-EPR (250 MHz)	78	-No hypoxia: 14<br>-Hypoxic fraction > 0.5: 3<br>-Experimental failure in the process of radiation: 7	54
MCa4 mammary adenocarcinomas	LF-EPR (250 MHz)	65	-No hypoxia: 1<br>-Experimental failure in the process of radiation: 16	48
SCC7 squamous cell carcinomas	LF-EPR (250 MHz)	70	-No hypoxia: 1<br>-Hypoxic fraction > 0.4: 6<br>-Experimental failure in the process of radiation: 15	44
SCC7 squamous cell carcinomas	JIVA-25 (720 MHz)	48	-No hypoxia: 1<br>-Hypoxic fraction > 0.4: 1<br>-Experimental failure in the process of radiation: 6<br>-Experimental failure in EPR imaging: 2	38

Supplementary Table S2: A complete table with the data for all experiments conducted in this study: tumor type, treatment type, the number of days the tumors were measured (until recurrence or end of observation period of 90-day for FSA and 180-day for all other experiments), the hypoxic fraction at 10 torr (HF10), and the status of the tumor for Kaplan-Meier statistical analysis (regression coded as 1, recurred coded as 0, or censored coded as 2).

Tumor Type	EPR imager	Subject	Boost Treatment (0=Oxygenated; 1=Hypoxic)	Time to Recurrence (days)	HF10	Volume (mm^3)	Delay between imaging and RT (hours)	Status (0=Recurred; 1=Cured; 2=Censored)
SCC7	250-MHz	1	1	180	0.033	351	2.87	1
SCC7	250-MHz	2	0	180	0.157	322	2.02	1
SCC7	250-MHz	3	1	180	0.346	357	2.60	1
SCC7	250-MHz	4	1	180	0.279	256	3.43	1
SCC7	250-MHz	5	0	57	0.312	426	1.68	0
SCC7	250-MHz	6	1	39	0.001	411	3.20	0
SCC7	250-MHz	7	0	168	0.252	482	5.12	0
SCC7	250-MHz	8	1	180	0.056	287	3.97	1
SCC7	250-MHz	9	1	180	0.117	490	2.52	1
SCC7	250-MHz	10	1	41	0.131	293	6.10	2
SCC7	250-MHz	11	0	180	0.211	408	3.08	1
SCC7	250-MHz	12	1	180	0.209	326	4.67	1
SCC7	250-MHz	13	0	180	0.021	252	4.92	1
SCC7	250-MHz	14	1	91	0.080	290	4.72	0
SCC7	250-MHz	15	0	180	0.299	281	5.83	1
SCC7	250-MHz	16	1	180	0.399	323	4.20	1
SCC7	250-MHz	17	0	39	0.198	286	6.00	0
SCC7	250-MHz	18	0	36	0.310	368	6.08	0
SCC7	250-MHz	19	1	115	0.319	343	2.87	0
SCC7	250-MHz	20	1	76	0.390	301	3.07	0
SCC7	250-MHz	21	0	86	0.253	474	4.50	0
SCC7	250-MHz	22	0	62	0.366	469	3.97	0
SCC7	250-MHz	23	1	180	0.281	348	3.13	1
SCC7	250-MHz	24	1	117	0.306	256	2.63	0
SCC7	250-MHz	25	0	41	0.196	292	4.60	0
SCC7	250-MHz	26	1	180	0.210	284	2.55	1
SCC7	250-MHz	27	0	45	0.395	353	3.63	0
SCC7	250-MHz	28	1	70	0.394	271	2.15	0
SCC7	250-MHz	29	0	180	0.140	280	1.90	1
SCC7	250-MHz	30	1	180	0.242	371	1.95	1
SCC7	250-MHz	31	0	180	0.223	212	1.92	1

SCC7	250-MHz	32	1	74	0.185	437	2.52	0
SCC7	250-MHz	33	1	30	0.081	264	1.73	2
SCC7	250-MHz	34	0	78	0.132	348	2.85	2
SCC7	250-MHz	35	0	62	0.276	428	2.05	0
SCC7	250-MHz	36	1	180	0.224	349	2.67	1
SCC7	250-MHz	37	0	104	0.143	287	2.35	0
SCC7	250-MHz	38	0	134	0.094	428	1.85	2
SCC7	250-MHz	39	0	180	0.313	439	1.85	1
SCC7	250-MHz	40	0	22	0.241	424	1.28	2
SCC7	250-MHz	41	0	9	0.152	353	1.55	2
SCC7	250-MHz	42	1	67	0.213	352	1.53	0
SCC7	250-MHz	43	1	59	0.323	383	2.20	0
SCC7	250-MHz	44	1	180	0.099	358	3.55	1
SCC7	720-MHz	1	1	24	0.250	405	3.58	2
SCC7	720-MHz	2	1	88	0.145	423	3.72	2
SCC7	720-MHz	3	0	81	0.049	239	2.55	0
SCC7	720-MHz	4	1	87	0.050	346	3.45	2
SCC7	720-MHz	5	1	67	0.156	488	4.40	0
SCC7	720-MHz	6	0	56	0.073	490	3.60	0
SCC7	720-MHz	7	0	72	0.101	397	3.45	0
SCC7	720-MHz	8	1	180	0.124	353	4.77	1
SCC7	720-MHz	9	1	180	0.150	379	3.37	1
SCC7	720-MHz	10	1	180	0.317	278	3.65	1
SCC7	720-MHz	11	0	65	0.177	395	4.17	0
SCC7	720-MHz	12	0	61	0.217	379	2.53	0
SCC7	720-MHz	13	0	40	0.126	476	3.13	0
SCC7	720-MHz	14	1	121	0.195	428	4.52	0
SCC7	720-MHz	15	1	180	0.153	318	2.70	1
SCC7	720-MHz	16	1	180	0.092	385	2.05	1
SCC7	720-MHz	17	0	60	0.163	321	3.08	0
SCC7	720-MHz	18	1	180	0.222	359	3.43	1
SCC7	720-MHz	19	1	180	0.140	366	2.92	1
SCC7	720-MHz	20	0	54	0.279	290	1.98	0
SCC7	720-MHz	21	1	180	0.143	308	2.43	1
SCC7	720-MHz	22	0	115	0.161	275	4.85	0
SCC7	720-MHz	23	0	180	0.274	293	2.80	1
SCC7	720-MHz	24	1	75	0.245	467	3.00	0
SCC7	720-MHz	25	0	180	0.194	272	1.95	1
SCC7	720-MHz	26	0	50	0.362	375	1.67	0

SCC7	720-MHz	27	1	169	0.362	305	1.63	1
SCC7	720-MHz	28	1	59	0.216	236	1.88	0
SCC7	720-MHz	29	1	163	0.124	277	2.73	1
SCC7	720-MHz	30	0	49	0.119	307	1.87	0
SCC7	720-MHz	31	1	27	0.130	417	2.75	2
SCC7	720-MHz	32	0	143	0.320	336	1.70	1
SCC7	720-MHz	33	1	76	0.102	233	1.72	0
SCC7	720-MHz	34	1	180	0.074	366	1.72	1
SCC7	720-MHz	35	1	180	0.023	350	1.58	1
SCC7	720-MHz	36	0	180	0.121	290	1.78	1
SCC7	720-MHz	37	0	180	0.302	359	1.35	1
SCC7	720-MHz	38	0	180	0.294	374	3.15	1
MCa4	250-MHz	1	1	180	0.089	214	1.32	1
MCa4	250-MHz	2	1	5	0.104	345	1.37	2
MCa4	250-MHz	3	1	180	0.395	344	1.72	1
MCa4	250-MHz	4	1	83	0.096	369	1.38	0
MCa4	250-MHz	5	1	180	0.018	338	1.50	1
MCa4	250-MHz	6	1	180	0.035	313	1.40	1
MCa4	250-MHz	7	1	180	0.373	416	1.27	1
MCa4	250-MHz	8	0	180	0.362	353	1.50	1
MCa4	250-MHz	9	1	180	0.097	357	2.53	1
MCa4	250-MHz	10	1	180	0.338	432	2.88	1
MCa4	250-MHz	11	0	180	0.343	347	4.48	1
MCa4	250-MHz	12	0	180	0.367	380	5.95	1
MCa4	250-MHz	13	0	180	0.138	308	4.10	1
MCa4	250-MHz	14	0	180	0.153	324	4.98	1
MCa4	250-MHz	15	0	133	0.196	257	4.55	0
MCa4	250-MHz	16	0	43	0.078	305	5.98	0
MCa4	250-MHz	17	0	180	0.176	296	5.28	1
MCa4	250-MHz	18	1	180	0.306	267	4.62	1
MCa4	250-MHz	19	0	51	0.111	397	3.73	0
MCa4	250-MHz	20	1	57	0.144	307	3.55	0
MCa4	250-MHz	21	1	55	0.018	318	3.47	0
MCa4	250-MHz	22	0	68	0.105	360	2.68	0
MCa4	250-MHz	23	1	120	0.256	430	4.13	0
MCa4	250-MHz	24	0	50	0.148	304	3.55	0
MCa4	250-MHz	25	1	48	0.037	256	2.77	0
MCa4	250-MHz	26	0	44	0.094	385	3.20	0
MCa4	250-MHz	27	1	180	0.051	328	3.15	1
MCa4	250-MHz	28	0	83	0.130	294	2.70	0

MCa4	250-MHz	29	1	84	0.058	259	3.45	0
MCa4	250-MHz	30	0	138	0.145	355	3.22	0
MCa4	250-MHz	31	1	180	0.294	380	3.62	1
MCa4	250-MHz	32	0	158	0.167	283	2.70	0
MCa4	250-MHz	33	1	180	0.021	372	5.05	1
MCa4	250-MHz	34	0	54	0.194	368	3.65	0
MCa4	250-MHz	35	1	180	0.219	385	3.20	1
MCa4	250-MHz	36	0	72	0.092	315	4.22	0
MCa4	250-MHz	37	1	180	0.185	390	3.53	1
MCa4	250-MHz	38	0	180	0.134	448	3.08	1
MCa4	250-MHz	39	1	180	0.174	340	4.20	1
MCa4	250-MHz	40	1	180	0.355	395	5.50	1
MCa4	250-MHz	41	0	43	0.049	418	3.67	0
MCa4	250-MHz	42	0	53	0.140	330	4.42	0
MCa4	250-MHz	43	1	180	0.120	328	3.33	1
MCa4	250-MHz	44	0	61	0.029	372	5.67	0
MCa4	250-MHz	45	1	38	0.007	361	3.63	0
MCa4	250-MHz	46	1	84	0.230	303	3.03	0
MCa4	250-MHz	47	0	63	0.023	305	4.33	0
MCa4	250-MHz	48	1	180	0.348	277	3.48	1
FSa	250-MHz	1	1	10	0.169	599	1.48	2
FSa	250-MHz	2	0	90	0.048	355	1.63	1
FSa	250-MHz	3	1	90	0.034	271	1.30	1
FSa	250-MHz	4	0	38	0.044	271	1.43	0
FSa	250-MHz	5	1	90	0.505	355	0.98	1
FSa	250-MHz	6	0	17	0.281	301	1.27	2
FSa	250-MHz	7	1	38	0.227	255	1.22	0
FSa	250-MHz	8	0	37	0.142	483	1.28	0
FSa	250-MHz	9	1	90	0.055	553	0.93	1
FSa	250-MHz	10	1	35	0.159	520	1.25	0
FSa	250-MHz	11	1	20	0.008	384	1.08	2
FSa	250-MHz	12	1	90	0.328	432	1.27	1
FSa	250-MHz	13	0	68	0.057	415	1.33	0
FSa	250-MHz	14	1	33	0.036	381	1.18	2
FSa	250-MHz	15	0	21	0.055	311	0.95	0
FSa	250-MHz	16	1	90	0.306	366	0.98	1
FSa	250-MHz	17	1	23	0.488	414	1.10	2
FSa	250-MHz	18	0	31	0.081	296	1.13	0
FSa	250-MHz	19	1	21	0.019	334	0.95	0

FSa	250-MHz	20	0	21	0.153	476	0.95	0
FSa	250-MHz	21	1	90	0.307	438	1.03	1
FSa	250-MHz	22	0	21	0.219	702	1.35	0
FSa	250-MHz	23	1	11	0.488	437	1.22	2
FSa	250-MHz	24	0	20	0.359	373	1.10	2
FSa	250-MHz	25	0	15	0.123	436	1.10	0
FSa	250-MHz	26	1	24	0.08	437	1.07	0
FSa	250-MHz	27	0	12	0.09	486	1.13	2
FSa	250-MHz	28	1	21	0.005	391	1.49	0
FSa	250-MHz	29	1	90	0.28	394	1.43	1
FSa	250-MHz	30	0	19	0.044	361	1.55	0
FSa	250-MHz	31	1	44	0.244	571	1.40	2
FSa	250-MHz	32	0	19	0.215	520	1.57	2
FSa	250-MHz	33	1	42	0.311	367	1.40	2
FSa	250-MHz	34	0	26	0.162	385	1.33	2
FSa	250-MHz	35	1	90	0.243	346	1.15	1
FSa	250-MHz	36	1	90	0.261	533	1.27	1
FSa	250-MHz	37	1	25	0.013	356	1.00	0
FSa	250-MHz	38	0	90	0.032	328	0.98	1
FSa	250-MHz	39	0	90	0.135	485	1.17	1
FSa	250-MHz	40	0	21	0.286	743	1.68	2
FSa	250-MHz	41	1	32	0.017	374	4.62	0
FSa	250-MHz	42	0	39	0.084	408	1.97	0
FSa	250-MHz	43	0	17	0.12	413	1.20	0
FSa	250-MHz	44	1	17	0.016	357	1.28	0
FSa	250-MHz	45	1	90	0.132	305	1.17	1
FSa	250-MHz	46	1	90	0.153	386	1.23	1
FSa	250-MHz	47	0	26	0.283	400	1.08	0
FSa	250-MHz	48	1	90	0.088	390	1.23	1
FSa	250-MHz	49	0	20	0.153	378	1.16	0
FSa	250-MHz	50	1	22	0.019	308	1.48	0
FSa	250-MHz	51	0	25	0.18	397	1.48	0
FSa	250-MHz	52	1	33	0.045	341	1.37	0
FSa	250-MHz	53	0	90	0.304	524	1.53	1
FSa	250-MHz	54	0	90	0.107	470	1.53	1
